# Clinical predictors, microbiological profile, and outcomes of bacteremia in patients with cellulitis: a 6-year retrospective cohort study

**DOI:** 10.1007/s10096-026-05593-3

**Published:** 2026-07-13

**Authors:** Ana Cotos Suárez, Amparo López-Bernus, Montserrat Alonso-Sardón, Virginia Velasco Tirado, Laura Sancha Domínguez, Beatriz Rodriguez Alonso, Josue Pendones Ulerio, Juan Luis Muñoz Bellido, Moncef Belhassen-García

**Affiliations:** 1 Servicio de Microbiología y Parasitología, Hospital Universitario de Salamanca (HUS), Instituto de Investigación Biomédica de Salamanca (IBSAL), Centro de Investigación en Enfermedades Tropicales (CIETUS), Universidad de Salamanca, Salamanca, Spain; 2 Servicio de Medicina Interna, Unidad de Infecciosas. HUS. IBSAL. CIETUS, Universidad de Salamanca, Salamanca, Spain; 3 Área de Medicina Preventiva, Epidemiología y Salud Pública, Facultad de Medicina, CIETUS, IBSAL, Universidad de Salamanca, Salamanca, Spain; 4 Servicio de Dermatologia, HUS, IBSAL, CIETUS, Universidad de Salamanca, Salamanca, Spain; 5 Servicio de Microbiología y Parasitología, HUS, IBSAL, CIETUS, Departamento de Ciencias Biomédicas y del Diagnóstico, Universidad de Salamanca, CSIC, Salamanca, Spain; 6 Servicio de Medicina Interna, Unidad de Infecciosas, HUS, IBSAL, E-INTRO, CIETUS, Universidad de Salamanca, Salamanca, Spain

**Keywords:** Cellulitis, Bacteremia, Blood cultures, CREST classification

## Abstract

**Background:**

Cellulitis is a common cause of hospital admission, yet the clinical relevance and predictors of bacteremia remain incompletely characterized. Identifying patients at risk of bloodstream infection may help optimize diagnostic strategies and clinical management.

**Methods:**

We conducted a retrospective observational study at a tertiary-care hospital including all consecutive patients hospitalized with cellulitis between January 2019 and December 2024. Patients without blood cultures were excluded. Clinical severity was stratified using the CREST classification. Microbiological data, antibiotic therapy, hospital outcomes, and follow-up at 1 and 3 months were analyzed.

**Results:**

Among 311 episodes included, bacteremia was confirmed in 46 cases (14.8%). Patients with bacteremia showed significantly higher clinical severity at admission, with CREST grades III–IV present in 56.5% compared with 36.6% in non-bacteremia patients (*p* < 0.001). Sepsis occurred in 65.2% of bacteremia cases versus 24.5% of non-bacteremia cases (*p* < 0.001). Bacteremia was associated with longer hospital stay (18 ± 18.3 vs 13 ± 9.7 days; *p* = 0.003) and more frequent antibiotic modifications during admission (*p* = 0.023). Streptococcal species and *Staphylococcus aureus* showed a higher propensity for bloodstream invasion. In-hospital mortality was higher among bacteremia patients (13.0% vs 7.9%), although the difference was not statistically significant. Three-month mortality reached 14.3% in the bacteremia group compared with 9.9% in non-bacteremia patients.

**Conclusion:**

Bacteremia complicating cellulitis is uncommon but associated with greater clinical severity, longer hospitalization, and increased therapeutic complexity. Severity stratification tools such as CREST may assist in identifying patients at increased risk of bacteremia; however, given their limited discriminatory performance, they should not be used in isolation to guide blood culture collection or other clinical decisions.

## Introduction

Cellulitis is one of the most common skin and soft tissue infections (SSTIs) worldwide, accounting for a significant proportion of emergency department visits and hospital admissions. In most cases, the course of the disease is mild, presenting local manifestations such as erythema and edema. However, complications may arise, including ulceration, abscess formation, or progression to bacteremia, which, if inadequately treated, can lead to severe systemic outcomes such as sepsis or necrotizing fasciitis [1]. The reported incidence of bacteremia in patients with cellulitis varies between 2% and 21.3%, depending on the study, clinical setting, and patient population [2].

The primary causative pathogen of cellulitis remains a subject of debate. Some studies identify β-hemolytic streptococci**,** particularly group A streptococci, as the predominant etiological agent [3], whereas others highlight *Staphylococcus aureus* as the main pathogen [4]; but there is widespread consensus that streptococci are the most common microorganisms in cellulitis-related bacteremia [1, 2].

Currently, the global incidence of cellulitis is estimated at 200 cases per 100,000 population [1]. The condition accounts for up to 14% of emergency department visits [5] and more than 10% of hospital admissions [1]. Furthermore, the presence of bacteremia considerably prolongs patients’ hospital stays [6], so early identification of the causative pathogen is crucial for optimizing treatment, guiding admission decisions, and preventing severe complications such as sepsis.

The primary objective of this study was to analyze the incidence, clinical predictors, and microbiological etiology of bacteremia in a cohort of hospitalized patients with cellulitis over a six-year period (2019–2024). Furthermore, we aimed to evaluate the impact of bacteremia on hospital management and clinical resolution at one and three months post-discharge, providing a comprehensive outlook on the prognostic value of bloodstream invasion in this population. To our knowledge, this is one of the few studies integrating clinical severity scores, microbiological data, and medium-term outcomes in a real-world cohort of hospitalized cellulitis patients.

## Material and methods

### Study design and setting

We conducted a retrospective observational study at the Hospital Universitario de Salamanca (Castilla y León, Spain), a tertiary-care teaching hospital with 863 beds. The study period included all patients hospitalized with a diagnosis of cellulitis and blood culture obtained between January 2019 and December 2024, who were diagnosed by the internal medicine department.

### Participants and inclusion criteria

Patients were selected from the hospital database using ICD-10 criteria; specifically, those diagnosed with cellulitis (L03 group). Next, the medical records were reviewed to confirm that the patients were indeed suffering from cellulitis and to determine its cause. Only those who were diagnosed by a consultant in internal medicine, absence of abscesses or pus near to the cellulitis at the moment of admission, and blood cultures taken were included. To ensure a consistent comparison between bacteremic and non-bacteremic cases, only patients in whom blood cultures were obtained at the time of cellulitis diagnosis were considered. Blood cultures were obtained at the discretion of the treating physician, based on clinical severity, suspicion of systemic infection, and risk of immunosuppression. Consequently, patients without blood cultures were excluded, which may introduce a selection bias toward more clinically severe cases in which blood cultures were deemed clinically indicated. The unit of analysis was the cellulitis episode; therefore, patients with more than one episode during the study period were included as independent cases.

Cellulitis was diagnosed on the basis of the patient’s medical history and physical examination regardless of the use of other microbiological or imaging diagnostic procedures [7–9]. Patients with cellulitis as either a primary or secondary diagnosis were included. Cellulitis as a primary diagnosis means that the patient had only cellulitis at the time of admission. Cellulitis as a secondary diagnosis refers to that which developed during the hospital stay or was detected during the medical examination due to another condition. In the latter case, the blood cultures included in the study are those taken at the time of diagnosis of cellulitis, in patients who had not previously positive blood cultures during their hospital stay.

### Clinical severity stratification and risk assessment

The severity of each cellulitis episode was stratified using the Clinical Resource Efficiency Support Team (CREST) criteria, integrated with the Standardized Early Warning Score (SEWS) [10–13], to categorize patients based on their physiological status and risk of deterioration. These scales classify cellulitis into four grades according to systemic involvement and comorbidities:

- Grades I–II: Localized infection without controlled comorbidities. SEWS < 4
- Grade II: Localized infection with controlled comorbidities. SEWS < 4
- Grade III: Systemic signs of infection and unstable comorbidities but SEWS < 4
- Grade IV: Systemic signs of infection and unstable comorbidities but SEWS > 4

The Quick Sepsis-related Organ Failure Assessment (qSOFA) [14] and the National Early Warning Score 2 (NEWS2) [15] indices were used to assess the potential for risk stratification of bacteraemia following Rath et al. algorithm [16].

Sepsis was considered if the patient has complied with the parameters of the diagnostic criteria of the Third International Consensus Definitions for Sepsis and Septic shock [14].

### Microbiological analysis

Samples from the infected area were obtained by needle puncture, biopsy, or surface swab when invasive methods were unfeasible. Blood cultures were collected via peripheral venipuncture and incubated in the BD Bactec™ FX system (Becton Dickinson, USA) for a maximum of 5 days. Both samples from the infected area and blood culture samples were seeded on 5% sheep blood, chocolate, and MacConkey agars in aerobic atmosphere with 5% CO_2_ and anaerobic atmosphere at 37 °C. Microorganism identification was performed using Matrix-Assisted Laser Desorption/Ionization-Time of Flight mass spectrometry (MALDI-TOF MS) (Biotyper®, Bruker Daltonics, Germany).

### Follow-up and outcome measures

The clinical evolution of patients was analyzed at one and three months post-discharge using electronic medical records to corroborate the outcome of the infection. Outcomes were classified as clinical resolution, unresolved infection, hospital readmission, or all-cause mortality.

Resolved cellulitis means cure without any complications. Unresolved cellulitis means that the infection became worse or not cured in the 30 following days of discharge.

### Statistical analysis

Statistical analysis was performed using SPSS version 30.0. Qualitative variables are presented as absolute frequency distributions and percentages. Quantitative variables are expressed as mean ± standard deviation (SD) or median and interquartile range (IQR). Categorical variables were compared using the χ^2^ test or Fisher’s exact test, as appropriate. Continuous variables were compared using one-way ANOVA or the Mann–Whitney U test, depending on data distribution. Statistical significance was set at *p* < 0.05. Missing data were handled using complete-case analysis, and no imputation methods were applied. Receiver operating characteristic (ROC) curve analysis was performed to assess the ability of the CREST, qSOFA, and NEWS2 scores to discriminate between bacteremic and non-bacteremic patients. The area under the curve (AUC) with 95% confidence intervals (95% CI) was calculated, and optimal cut-off values were determined using the Youden index.

### Ethics committee

This study was approved by the Institutional Ethics Committee of the CAUSA (Reference: CEIm PI 2023 07 1392). All data were anonymized by assigning unique identification codes to each patient. The study was conducted in accordance with the ethical standards of the Declaration of Helsinki, revised in 2024 [17].

## Results

### Study population and baseline characteristics

During the six-year study period (2019–2024), a total of 817 patients were hospitalized at the University Hospital of Salamanca with a clinical diagnosis of cellulitis. Blood cultures were performed in 311 cases (311/817, 38.1%), which constituted the final study population for this analysis. Among the tested patients, bacteremia was confirmed in 46 cases, yielding a bacteremia rate of 14.8% (46/311) among tested patients and 5.6% (46/817) among the total cohort of hospitalized patients.

The mean age of the study cohort (*n* = 311) was 67 ± 23.5 years, and the sex distribution was balanced, with 156 men (156/311, 50.2%) and 155 women (155/311, 49.8%). As detailed in Table 1, no statistically significant differences were observed between the bacteremia and non-bacteremia groups regarding age or sex (*P* > 0.05).

**Table 1:** Baseline clinical characteristics of hospitalized patients with cellulitis: comparison according to bacteremia status

Variable	Total episodes *N* = 311	Non-bacteremia *n* = 265	Bacteremia *n* = 46	*P-value*
Sociodemographic variables	n (%)	n (%)	n (%)	
Age, years (mean ± SD)	67 ± 23.5	69 ± 21.7	66 ± 23.8	0.507
Male sex	156 (50.2%)	134 (50.6%)	22 (47.8%)	0.732
Female sex	155 (49.8%)	131 (49.4%)	24 (52.2%)	
Diagnosis				
Principal diagnosis of cellulitis	151 (48.6%)	131 (49.4%)	20 (43.5%)	0.456
Secondary diagnosis of cellulitis	160 (51.4%)	134 (50.6%)	26 (56.5%)	
• Nosocomial	27/160 (16.9%)	23/134 (17.2%)	4/26 (15.4%)	0.827
Predisposing factors				
Prior ulcer	51 (16.4%)	41 (15.5%)	10 (21.7%)	0.262
Prior wound	75 (24.1%)	66 (24.9%)	9 (19.6%)	
• Surgical wound	30 (9.6%)	26 (9.8%)	4 (8.7%)	
• Animal bites	3 (1.0%)	2 (0.8%)	1 (2.2%)	
• Insect bites	4 (1.3%)	1 (0.4%)	3 (6.5%)	
• Other wounds	45 (14.5%)	40 (15.1%)	5 (10.9%)	
Prior trauma	33 (10.6%)	29 (10.9%)	4 (8.7%)	
Lymphedema	13 (4.2%)	10 (3.8%)	3 (6.5%)	
Unresolved/prior cellulitis	17 (5.5%)	15 (5.7%)	2 (4.3%)	
Severity of cellulitis (CREST^*^ classification)				
I	4 (1.3%)	3 (1.1%)	1 (2.2%)	< 0.001
II	184 (59.1%)	165 (62.3%)	19 (41.3%)	
III	72 (23.2%)	61 (23.0%)	11 (23.9%)	
IV	51 (16.4%)	36 (13.6%)	15 (32.6%)	
Location of cellulitis				
Lower extremities	221 (71.3%)	185 (70.1%)	36 (78.3%)	0.303
Upper extremities	32 (10.3%)	26 (9.8%)	6 (13.0%)	
Trunk	35 (11.3%)	33 (12.5%)	2 (4.3%)	
Head/neck	22 (7.1%)	20 (7.6%)	2 (4.3%)	
Sepsis	95 (30.5%)	65 (24.5%)	30 (65.2%)	< 0.001
Comorbidities				
Hypertension	159 (51.1%)	132 (49.8%)	27 (58.7%)	0.266
Chronic and post-cancer lymphedema/chronic vascular disease	100 (32.2%)	86 (32.5%)	14 (30.4%)	0.787
Immunosuppression/Malignancy	85 (27.3%)	68 (25.7%)	17 (37.0%)	0.113
Diabetes mellitus	75 (24.1%)	65 (24.5%)	10 (21.7%)	0.683
Obesity	49 (15.8%)	46 (17.4%)	3 (6.5%)	0.063
Alcohol use disorder & smoking	45 (14.5%)	37 (14.0%)	8 (17.4%)	0.542
Hospital discharge service				
Internal medicine	175 (56.3%)	155 (58.5%)	20 (43.5%)	0.212
Hematology	27 (8.7%)	21 (7.9%)	6 (13.0%)	
Pediatrics	19 (6.1%)	17 (6.4%)	2 (4.3%)	
Dermatology	18 (5.8%)	16 (6.0%)	2 (4.3%)	
Traumatology	16 (5.1%)	14 (5.3%)	2 (4.3%)	
Angiology/Vascular Surgery	8 (2.6%)	6 (2.3%)	2 (4.3%)	
Other surgeries	7 (2.2%)	7 (2.6%)	0	

Continuous variables were compared using one-way ANOVA and categorical variables using the χ^2^ test or Fisher’s exact test, as appropriate. *CREST Clinical Resource Efficiency Support Team.

The most infected body part was the lower extremities, which accounted for 71.3% of all episodes, with no significant differences observed based on bacteremia status (*p* = 0.252). Clinical characteristics, including the primary versus secondary nature of the diagnosis and the presence of underlying comorbidities such as diabetes mellitus (75/311, 24.1%) and immunosuppression (85/311, 27.3%), are further described in Table 1. This approach reflects a clinically selected population, likely enriched for more severe presentations.

### Clinical predictors: severity

**Fig. 1:**
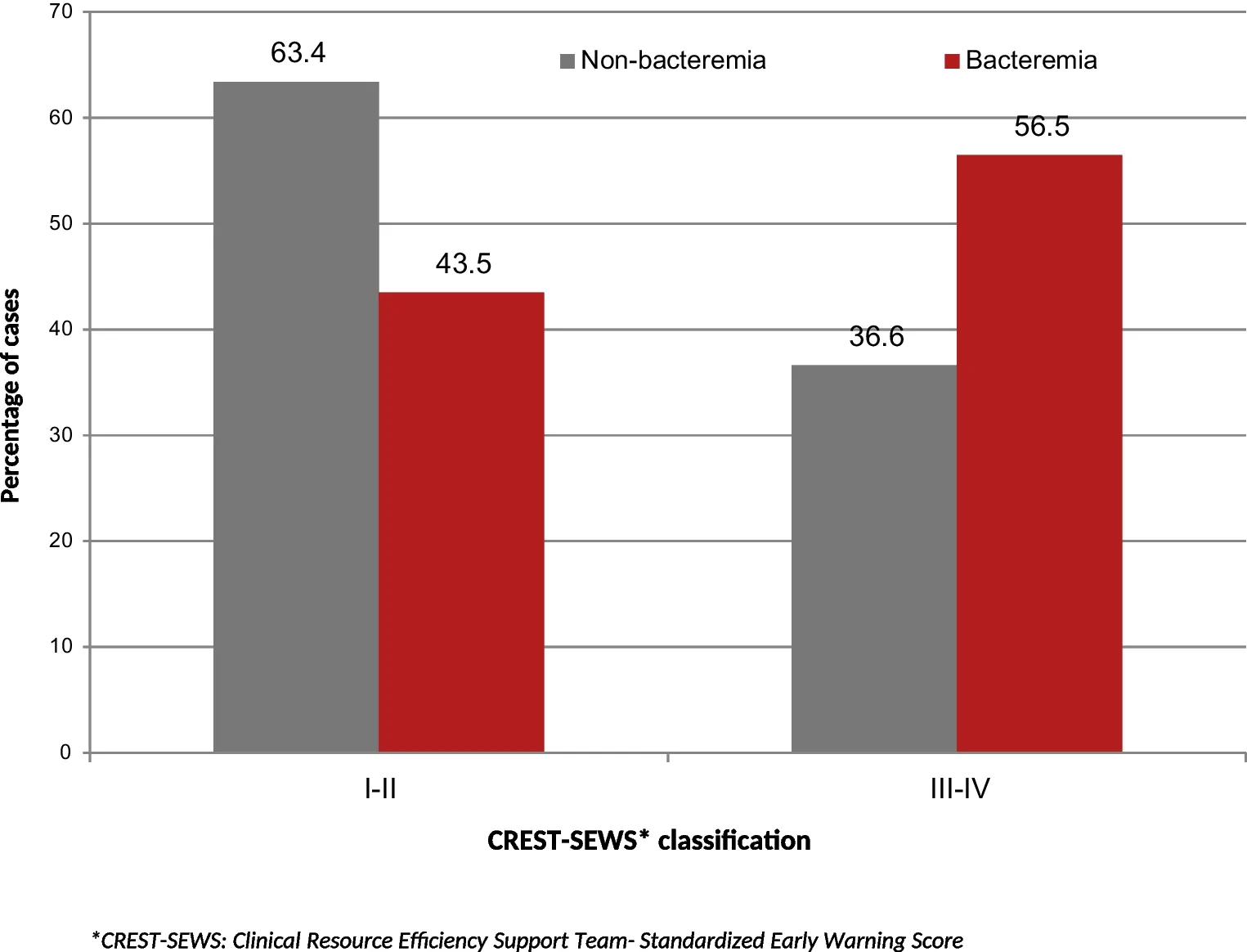
Distribution of cellulitis severity according to CREST classification by bacteremia status, *p* < 0.018. *CREST-SEWS: Clinical Resource Efficiency Support Team- Standardized Early Warning Score

The severity of cellulitis at admission, stratified using the CREST classification, was a significant predictor of bloodstream infection (*p* < 0.001, Table 1). Patients with higher clinical severity (CREST grades III–IV) had increased odds of bacteremia compared with those with lower severity (CREST grades I–II) (OR = 2.1, 95% CI 1.1–4.0; *p* = 0.018). Accordingly, severe presentations accounted for 26 of 46 bacteremia cases (56.5%), compared with 97 of 265 non-bacteremic episodes (36.6%) (Fig. 1). Notably, CREST grade IV was more than twice as frequent among patients with bacteremia than among those without bacteremia (15/46 [32.6%] vs. 36/265 [13.6%]).

Sepsis severity scores at admission were significantly higher among patients with bacteremia than among those without bloodstream infection (Table 2). The median CREST score was 3 (IQR 2–4) in bacteremic patients compared with 2 (IQR 2–3) in non-bacteremic patients (*P* < 0.001). Likewise, median qSOFA and NEWS2 scores were significantly higher in the bacteremia group, reaching 1 (IQR 0–2) versus 0 (IQR 0–1) and 5 (IQR 3–7) versus 3 (IQR 1–5), respectively (both *P* < 0.001). Mean values showed a similar pattern across all three severity scores.

**Table 2:** Comparison of sepsis severity scores according to bacteremia status and diagnostic performance for predicting bloodstream infection

Score		Bacteremia (*n* = 46)	No bacteremia (*n* = 244)	AUC (95% CI)	ROC p-value**	Optimal cut-off*	Sensitivityn/N (%)	Specificity (%)	*P*-value***
CREST	Median (IQR)	3 (2–4)	2 (2–3)	0.621 (0.526–0.715)	0.012	≥ 3	26/46	156/244	< 0.001
	Mean ± SD	2.91 ± 0.91	2.53 ± 0.75				56.5%	63.9%	
qSOFA	Median (IQR)	1 (0–2)	0 (0–1)	0.610 (0.515–0.706)	0.024	≥ 2	11/46	227/244	< 0.001
	Mean ± SD	0.89 ± 0.97	0.49 ± 0.66				23.9%	93.0%	
NEWS2	Median (IQR)	5 (3–7)	3 (1–5)	0.624 (0.535–0.713)	0.006	≥ 5	25/46	163/244	< 0.001
	Mean ± SD	5.20 ± 3.48	3.78 ± 2.62				54.3%	66.8%	

Values are presented as median (interquartile range [IQR]) and mean ± standard deviation (SD). * Optimal cut-off determined by the maximum Youden index obtained from ROC curve analysis. ** ROC *p*-values test whether the area under the curve differs significantly from 0.5. *** *P*-values correspond to comparisons between bacteremic and non-bacteremic patients using the Mann–Whitney U test.

**Fig. 2:**
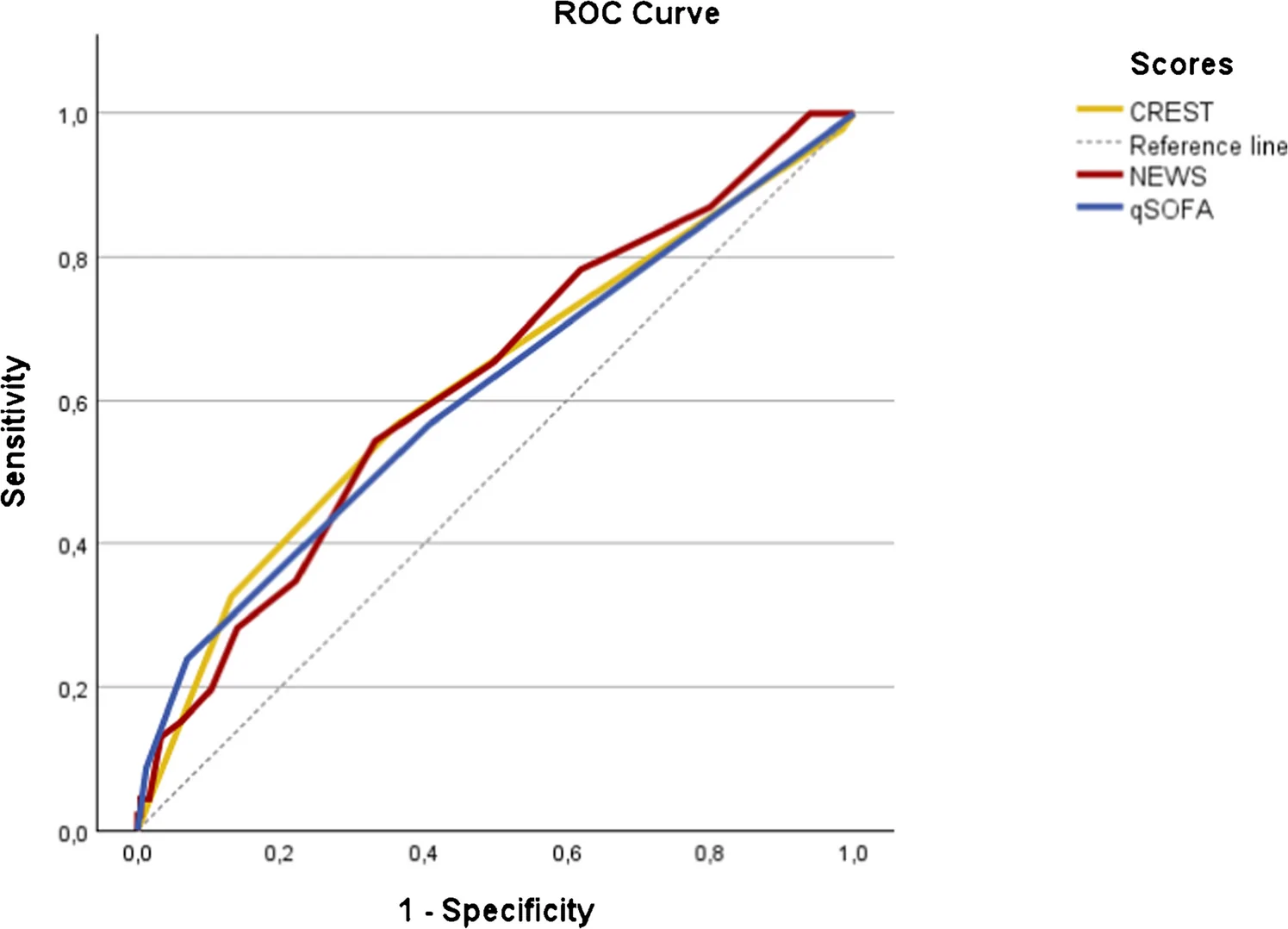
Receiver operating characteristic (ROC) curves of CREST, NEWS2 and qSOFA scores for predicting bacteremia in hospitalized patients with cellulitis

To evaluate their ability to identify bloodstream infection, ROC curve analyses were performed (Fig. 2). All three scores demonstrated statistically significant but limited discriminatory performance. The area under the curve (AUC) was 0.621 (95% CI 0.526–0.715) for CREST, 0.624 (95% CI 0.535–0.713) for NEWS2, and 0.610 (95% CI 0.515–0.706) for qSOFA (Table 2). NEWS2 showed the highest AUC, although differences between the three scores were minimal, and none achieved the threshold generally considered acceptable for clinical discrimination (AUC ≥ 0.70).

According to the maximum Youden index, the optimal cut-off value was ≥ 3 for CREST, yielding a sensitivity of 56.5% and a specificity of 63.9%. For NEWS2, the optimal cut-off was ≥ 5, providing a sensitivity of 54.3% and a specificity of 66.8%, whereas qSOFA ≥ 2 showed a sensitivity of 23.9% and a specificity of 93.0%. Thus, qSOFA offered the highest specificity but very low sensitivity, whereas CREST and NEWS2 provided a more balanced sensitivity–specificity profile. Nevertheless, all three scores demonstrated limited overall discrimination for identifying bloodstream infection in hospitalized patients with cellulitis. The ROC curves showed substantial overlap between bacteremic and non-bacteremic patients (Fig. 2).

### Temporal and microbiological trends

**Fig. 3:**
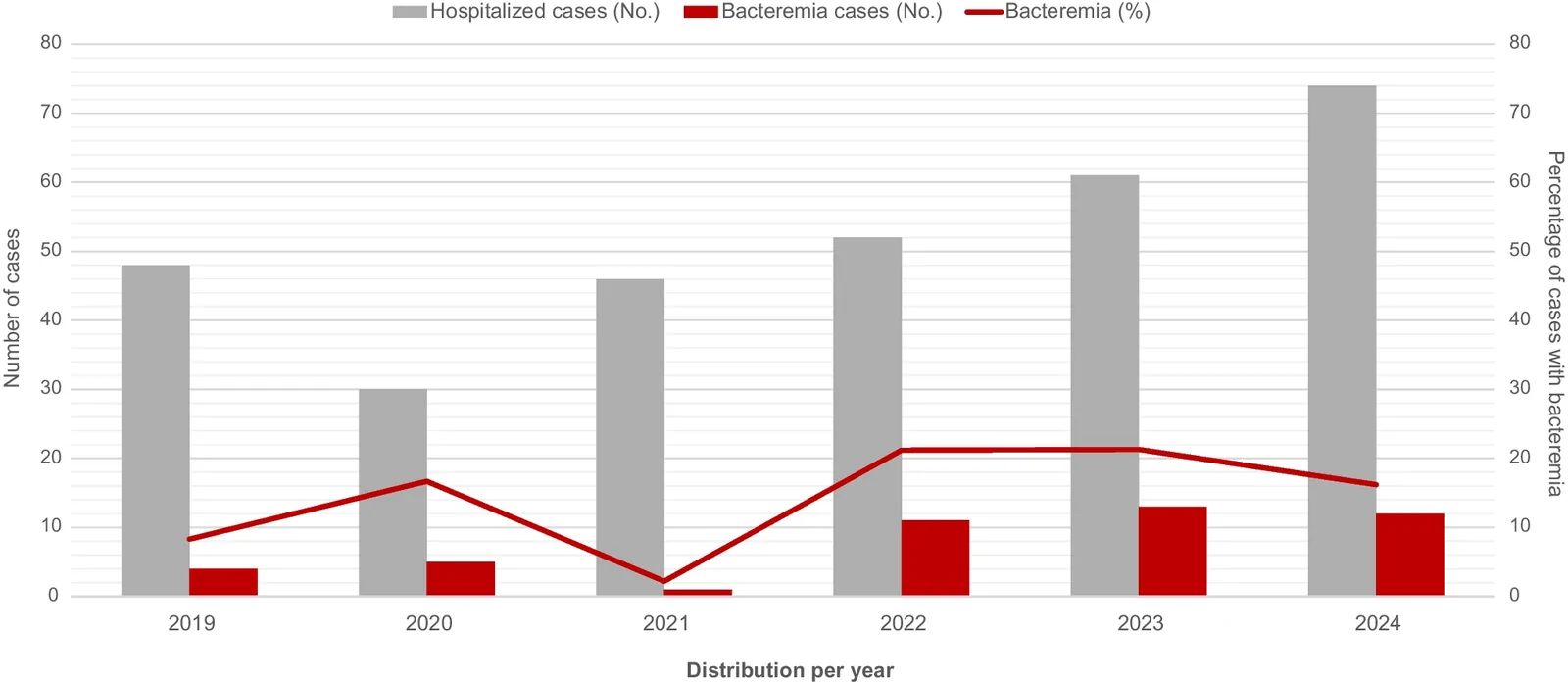
Annual distribution of hospitalized cellulitis patients and proportion of bacteremia during the study period (*p* = 0.045)

The annual distribution of hospitalized cellulitis cases and bacteremia incidence is shown in Fig. 3. The number of admissions increased progressively during the study period, from 48 cases in 2019 to 74 in 2024. The proportion of bacteremia showed significant annual variation (*p* = 0.045), with a notable increase beginning in 2022. The highest bacteremia incidence was observed in 2023 (21.3%), followed by 2022 (21.2%) and 2024 (16.2%), whereas lower proportions were recorded in the pre-pandemic years.

A total of 139 culture samples were collected (139/311, 44.7%), of which 81 were positive (81/139, 58.3%). Most positive cultures were monomicrobial (68/81, 84.0%), and the predominant microorganism was *S. aureus* (23/93 isolates, 24.7%). In the bacteraemia group, cultures were taken from 23 of 46 patients (50.0%), and 15 were positive (15/23, 65.2%), with *S. aureus* being the predominant microorganism (6/16 isolates, 37.5%) (Table 3).

**Table 3:** Microbiological characteristics of cellulitis cultures from the patients in the study

Variable	Total	Non-bacteremia	Bacteremia	P-value
Cellulitis culture	***n* = 311**	***n* = 265**	***n* = 46**	**0.887**
Yes	139 (44.7%)	116 (43.8%)	23 (50.0%)	
No	172 (55.3%)	149 (56.2%)	23 (50.0%)	
Result	***n* = 139**	***n* = 116**	***n* = 23**	**0.397**
Positive	81 (58.3%)	66 (56.9%)	15 (65.2%)	
Negative	58 (41.7%)	50 (43.1%)	8 (34.8%)	
Positive culture	***n* = 81**	***n* = 66**	***n* = 15**	**0.373**
Monomicrobial	68 (84.0%)	55 (83.3%)	13 (86.7%)	
Polymicrobial	13 (16.0%)	11 (16.7%)	2 (13.3%)	
Microorganisms	**(*n* = 93)**	**(*n* = 77)**	**(*n* = 16)**	**0.371**
* Staphylococcus aureus*	23 (24.7%)	17 (22.1%)	6 (37.5%)	
* Streptococcus* spp.	6 (6.5%)	5 (6.5%)	1 (6.3%)	
Other Gram-positive bacteria	10 (10.8%)	9 (11.7%)	1 (6.3%)	
- *Enterococcus* spp.	4 (36.4%)	3 (33.3%)	1 (100%)	
Gram negative bacilli	50 (53.8%)	43 (55.8%)	7 (43.8%)	
* - Pseudomonas aeruginosa*	10 (20.0%)	10 (23.3%)	0	
* - Klebsiella* spp.	10 (20.0%)	8 (18.6%)	2 (28.6%)	
- *Escherichia coli*	9 (18.0%)	7 (16.3%)	2 (28.6%)	
* - Enterobacter* spp.	5 (10.0%)	4 (9.3%)	1 (14.3%)	
* - Proteus* spp.	4 (8.0%)	3 (7.0%)	0	
* Candida* spp.	4 (4.3%)	3 (3.9%)	1 (6.3%)	

Percentages were calculated according to the denominator indicated in each section. Percentages for microorganism subcategories were calculated using the corresponding parent category as denominator. Statistical test used: χ.^2^

Among the 46 positive blood cultures, 2 were polymicrobial. *S. aureus* and *Streptococcus* spp. were the most common pathogens (12%, 25.0% respectively). Of the streptococci isolated, 5 were *S. pyogenes*, 5 were *S. agalactiae* and 2 were *S. dysgalactiae*. 37.5% [18] of the isolates were Gram-negative bacilli, of which 4 isolates were identified as *E. coli*, 3 as *K. pneumoniae*, 2 as *P. aeruginosa* and 2 as *Enterobacter* spp.

In 11 patients, the same microorganisms were isolated from both the blood and the cellulitis sample, with 2 of the cultures being polymicrobial. In these cases, the microorganisms isolated were *S. aureus* (5), *S. pyogenes* (1), gram-negative bacilli (5) and *C. glabrata* (1)**.** Of the 23 *S. aureus* isolates obtained from the cultures, 5 were Methicillin-resistant *Staphylococcus aureus* (MRSA). Only one MRSA was isolated from blood cultures.

### Hospital management and antibiotic therapy

The presence of bacteremia significantly impacted hospital resources utilization (Table 4).

**Table 4:** Clinical outcomes, follow-up, and antibiotic management according to bacteremia status

Variable	Total	Non-bacteremia	Bacteremia	*P-value*
Hospital outcomes	(*n* = 311)	(*n* = 265)	(*n* = 46)	
Length of stay, days (mean ± SD)	14 ± 11.5	13 ± 9.7	18 ± 18.3	0.003
ICU admission	2 (0.6%)	1 (0.4%)	1 (2.2%)	–
In-hospital mortality	27 (8.7%)	21 (7.9%)	6 (13.0%)	0.255
Follow-up at 1 month	**(*n* = 284)**	**(*n* = 244)**	**(*n* = 40)**	
Loss to follow-up	98/284 (25.0%)	84/244 (25.9%)	14/40 (20.0%)	0.175
Clinical control	213/284 (75.0%)	181/244 (74.1%)	32/40 (80.0%)	
• Resolved	133/213 (62.4%)	110/181 (60.8%)	23/32 (71.9%)	0.426
• Unresolved	68/213 (31.9%)	62/181 (34.3%)	6/32 (18.8%)	
• Deaths	12/213 (5.6%)	9/181 (5.0%)	3/32 (9.4%)	
Follow-up at 3 months	**(*n* = 272)**	**(*n* = 235)**	**(*n* = 37)**	
Loss to follow-up	72/272 (26.5%)	63/235 (26.8%)	9/37 (24.3%)	0.213
Clinical control	200/272 (73.5%)	172/235 (73.2%)	28/37 (75.7%)	
• Resolved	153/200 (76.5%)	132/172 (76.7%)	21/28 (75.0%)	0.656
• Unresolved	22/200 (11.0%)	20/172 (11.6%)	2/28 (7.1%)	
• Hospital readmission	4/200 (2.0%)	3/172 (1.7%)	1/28 (3.6%)	
• Deaths	21/200 (10.5%)	17/172 (9.9%)	4/28 (14.3%)	
Empirical treatment	**(*n* = 307)**	**(*n* = 261)**	**(*n* = 46)**	0.559
Amoxicillin/clavulanic acid	131 (42.7%)	118 (45.2%)	13 (28.3%)	
Piperacillin/tazobactam	61 (19.9%)	48 (18.4%)	13 (28.3%)	
Cephalosporins	46 (15.0%)	37 (14.2%)	9 (19.6%)	
Carbapenems	12 (3.9%)	10 (3.8%)	2 (4.3%)	
Other β-lactams	10 (3.3%)	8 (3.1%)	2 (4.3%)	
Fluoroquinolones	22 (7.2%)	19 (7.3%)	3 (6.5%)	
Lincosamides	9 (2.9%)	8 (3.1%)	1 (2.2%)	
Trimethoprim–sulfamethoxazole	9 (2.9%)	8 (3.1%)	1 (2.2%)	
Others	7 (2.3%)	5 (1.9%)	2 (4.3%)	
Treatment modification during admission	**(*n* = 307)**	**(*n* = 261)**	**(*n* = 46)**	**0.023**
No change	52 (16.9%)	46 (17.6%)	6 (13.0%)	
1 change	68 (22.1%)	64 (24.5%)	4 (8.7%)	
≥ 2 changes	187 (60.9%)	151 (57.9%)	36 (78.3%)	

Percentages were calculated using the denominator indicated for each section. Statistical test used: χ^2^.

The mean length of stay (LOS) was substantially higher for bacteremia patients (18 ± 18.3 days) compared to non-bacteremia patients (13 ± 9.7 days; *p* = 0.003).

Regarding pharmacological management, there were no significant differences in the choice of empirical treatment (*p* = 0.559), with ß-lactams being the most prescribed agents (47.4%). However, bacteremia patients required more complex therapeutic management; 78.3% (36/46) of them needed two or more treatment modifications during admission (*p* = 0.023), compared to 57.9% (151/261) in the non-bacteremia group (Table 4).

The distribution of antibiotic classes according to treatment line did not significantly differ between patients with and without bacteremia (*p* > 0.05) (Table 4). β-lactams, particularly amoxicillin/clavulanic acid and piperacillin/tazobactam, were the most prescribed empirical treatment in both groups. In patients with bacteremia, piperacillin–tazobactam was more frequently used as second-line therapy, reflecting the need for broader antimicrobial coverage in clinically severe cases. Despite similar initial antibiotic choices, bacteremia patients more frequently required treatment escalation or multiple antibiotic lines during hospitalization.

### Complications, follow-up and mortality

Sepsis occurred in 65.2% (30/46) of patients with bacteremia, whereas it was observed in only 24.5% (65/265) of those without bloodstream infection (Table 1). These findings highlight the close relationship between systemic inflammatory response and the likelihood of bloodstream dissemination in cellulitis.

The overall mortality and resolution rates are summarized in Table 4. In-hospital mortality was higher in the bacteremia group (6/46, 13%) than in the non-bacteremia group (21/265, 7.9%), although this did not reach statistical significance (*p* = 0.255). Of the 60 deaths, 14 were associated with cellulitis, all of which occurred during hospitalization. Of these patients, 4 died from sepsis caused by cellulitis.

By the 3-month follow-up, the cumulative all-cause mortality for the bacteremia group was 28.3%, showing a trend toward worse outcomes compared to the 17.7% observed in the non-bacteremia group (*p* = 0.095). Despite the higher initial severity, 75.0% of the surviving bacteremia patients achieved clinical resolution at three months.

Mortality was primarily associated with older age, disease severity, and systemic involvement. Patients aged ≥ 60 years accounted for most deaths (56/60, 93.3%) showing a strong association (OR 7.5 95%, CI 2.6–21.5; *p* < 0.001). Mortality was also significantly higher among patients in whom cellulitis was recorded as a secondary diagnosis rather than the principal cause of admission (OR 3.2, 95% CI 1.7–6.0; *p* < 0.001). Higher clinical severity at presentation, defined by CREST grades III–IV, was associated with an increased risk of death (OR 2., 95% CI 1.3–4.2 *p* = 0.003), as was the presence of sepsis during hospitalization (OR 1.8, 95% CI 1.0–3.3; *p* = 0.037). When stratified according to bacteremia status, similar clinical patterns were observed, but mortality appeared to be more strongly influenced by underlying clinical vulnerability, including immunosuppression or malignancy, although most associations did not reach statistical significance, likely due to the smaller number of deaths in this subgroup. Despite the lack of statistical significance, the consistent trend toward higher mortality in patients with bacteremia suggests clinical relevance (Table 5).

**Table 5:** Clinical factors associated with mortality in hospitalized cellulitis patients according to bacteremia status

Variables	Total deaths *N* = 60		Non-bacteremia *n* = 46		Bacteremia *n* = 14	
	*n* (%)	OR (95% CI) *p*-value	n (%)	OR (95% CI) *p*-value	*n* (%)	OR (95% CI) *p-*value
Sex						
Female	34 (56.7%)	1.4 (0.8–2.5) > 0.05	25 (54.3%)	1.2 (0.6–2.4) > 0.05	9 (64.3%)	2.3 (0.6–8.5) > 0.05
Male	26 (43.3%)		21 (45.7%)		5 (35.7%)	
Age						
≥ 60	56 (93.3%)	7.5 (2.6–21.5) < 0.001	43 (93.5%)	8.2 (2.5–27.4) < 0.001	13 (92.9%)	4.3 (0.5–38.5) > 0.05
< 60	4 (6.7%)		3 (6.5%)		1 (7.1%)	
Diagnosis						
Secondary diagnosis of cellulitis	44 (73.3%)	3.2 (1.7–6.0) < 0.001	36 (78.3%)	4.9 (2.3–10.3) < 0.001	8 (57.1%)	0.5 (0.1–1.9) > 0.05
Principal diagnosis of cellulitis	16 (26.7%)		10 (21.7%)		6 (42.9%)	
Severity of cellulitis (CREST classification)						
III–IV	34 (56.7%)	2.4 (1.3–4.2) 0.003	22 (47.8%)	2.0 (1.1–3.9) 0.029	12 (85.7%)	3.1 (0.6–16.6) > 0.05
I–II	26 (43.3%)		24 (52.2%)		2 (14.3%)	
Location of cellulitis						
Lower extremities	48 (80.0%)	1.8 (0.9–3.5) > 0.05	37 (80.4%)	1.9 (0.9–4.2) > 0.05	11 (78.6%)	1.0 (0.2–4.7) > 0.05
Others	12 (20.0%)		9 (19.6%)		3 (21.4%)	
Microorganism						
* S. aureus*	4 (17.4%)	-	2 (15.4%)	-	2 (20.0%)	-
* Streptococcus* spp.	2 (6.7%)		-		2 (20.0%)	
Gram-negative bacilli	17 (73.9%)		11 (84.6%)		6 (60.0%)	
Sepsis	25 (41.7%)	1.8 (1.0–3.3) 0.037	11 (23.9%)	1.5 (0.7–3.2) > 0.05	14 (100.0%)	-

*OR* odds ratio, *CI* confidence interval. Percentages are calculated within each subgroup. Statistical test used: χ.^2^

## Discussion

Beyond being viewed as an isolated microbiological finding, bacteremia in the context of cellulitis can be understood as a clinical phenotype that reflects a complex interaction between the infectious load, the host response and the presence of underlying comorbidities. The present study provides a comprehensive six-year analysis of bacteremia in hospitalized patients with cellulitis, highlighting important clinical predictors and patient outcomes.

Cellulitis is an acute spreading infection SSTI caused by the entry of a microorganism into the subcutaneous tissue and it course with local heat, edema, pain, and erythema [1]. It is usually mild but can worsen, leading to bacteraemia, which may in turn cause sepsis.

Bacteremia is a rare complication of cellulitis, with a highly variable prevalence depending on the study (between 2 and 21.3%) [2, 18–20]. In our study, we obtained a 14.8% rate of bacteremia when counting only patients from whom blood cultures were taken, and a 5.6% rate when counting all patients with cellulitis, so our data fall within this range. Although bacteremia was relatively infrequent in our cohort, its presence was found to be a significant predictor of severity, which is also associated with greater clinical complexity and increased use of healthcare resources.

Previous studies have shown that certain comorbidities, such as diabetes, alcohol abuse, cirrhosis, or lymphedema, the presence of an infected device may increase the risk of bacteremia [20, 21], so blood cultures should be taken in patients with these pathologies. In our case, it was observed that most patients with bacteremia were immunosuppressed (37.0%), had type II diabetes mellitus (21.7%) or had lymphedema (21.7%). Other factors such as advanced age [7, 21] are included in some studies, but in our study no differences were found in these cases.

Since the classification of the severity of skin and soft tissue infections proposed by Eron et al. [10], new proposals have been developed to refine it. Eron et al. assessed risk based on criteria of systemic toxicity, comorbidities and risk of clinical progression. However, although this system proved useful from a conceptual point of view, limitations were subsequently identified relating to the subjectivity of some criteria and their limited discriminatory power in certain clinical scenarios. Consequently, Marwick et al. [13] later developed the Dundee criteria, which incorporated more objective parameters derived from the Standardized Early Warning Score (SEWS) criteria and the early recognition of sepsis, allowing for a better correlation between the classification and the complexity of care required, and years later, Hashem et al. [11] confirmed the usefulness of SEWS parameters. Finally, Rath et al. [16] published a novel study focusing on non-purulent SSTIs, in which, instead of using the SEWS criteria to stratify risk, employed the qSOFA and NEWS criteria. However, some studies do not recommend the use of qSOFA in infections for risk stratification due to its low sensitivity [22]. Recently, Ritchie et al. [23], proposed their own early warning score, a change made by an employee in the UK, which has also proved useful. Likewise other studies like the one written by Cutfield et al. [24], ours provided relevant evidence on the applicability and reproducibility of these classification systems in routine cellulitis diagnostic, demonstrating that the Dundee classification can successfully predict key clinical outcomes (such as the need for blood cultures, longer hospital stays or higher all-cause mortality in the most severe cases).

Patients classified in higher severity categories (Grades III–IV) showed a significantly greater probability of developing bacteremia, and could therefore benefit from blood culture testing [21].

This conceptual framework has direct implications for the practical use of blood cultures, which should not be considered a routine practice, but as a stratification tool in scenarios of clinical uncertainty. In conclusion, the need to take blood cultures in cases of cellulitis is based on three factors [12]: the severity of the clinical picture (CREST III-IV), the risk of sepsis, and the patient immunity. This stratification can be carried out using various parameters, notably the qSOFA and EWS scores. As new methods for risk stratification are now available like qSOFA [14] and NEWS2 [15], we decided to compare these indices with the SEWS in the group of patients with positive bacteraemia following the model by Rath et al. [16]. Bacteremic patients presented significantly higher CREST, qSOFA, and NEWS2 scores at admission, reflecting greater clinical severity and physiological derangement. However, the discriminatory performance of these tools for identifying bloodstream infection was limited, with AUC values ranging from 0.610 to 0.624. Although NEWS2 achieved the highest AUC, differences between the three scores were minimal and none reached the threshold generally considered acceptable for clinical prediction. Interestingly, a CREST score ≥ 3 (corresponding to CREST III–IV severity) provided a sensitivity of 56.5% and a specificity of 63.9%, performing similarly to NEWS2 and better than qSOFA in terms of sensitivity. These findings suggest that severity scores may contribute to the overall clinical assessment of patients at increased risk of bacteremia, but their modest discriminatory performance indicates that they should not be used as stand-alone tools for blood culture collection or other clinical decision-making. The comparable performance of CREST and NEWS2 may indicate that cellulitis-specific severity assessment captures clinically relevant information not fully reflected by general sepsis-oriented scores.

It remains unclear to date which is the main causative agent of cellulitis. Some studies suggest that β-haemolytic streptococci are the most prevalent causative agents, and that *S. aureus* should be questioned as it is also found on the skin as part of the normal flora [3, 25, 26]. However, other authors maintain that *S. aureus* is the main agent isolated in this condition [4, 7]. In general, it has been observed that streptococci are more difficult to isolate in culture, but can be detected using other techniques, such as Polymerase chain reaction (PCR) [7, 25, 26]; for this reason, the role of *S. aureus* and Gram-negative bacilli in cellulitis is sometimes called into question. In this study, their infectious role can be confirmed in at least 10 isolates, since in 5 of the patients with a positive blood culture, *S. aureus* was isolated in both the blood culture and the sample, and in another 5, the same occurred with Gram-negative bacilli.

There is, however, a consensus that β-haemolytic streptococci are the main cause of bacteraemia associated with skin and soft tissue infections, and therefore of cellulitis [2, 7, 19–21, 26]. In our study, there were an equal number of isolates in the bacteraemias caused by *S. aureus* and by streptococci (25%), probably for the same reason explained above.

The high prevalence of *S. aureus* in our study is likely due to the origin of the samples, given that 24% and 16.4% of cellulitis cases stemmed from a wound or a pre-existing ulcer respectively, where this is the most common isolated microorganism [18]. Other bacteria isolated in high proportions were Gram-negative bacilli (37.5% of isolates in bacteremias and 53.8% of the microorganisms isolated in cellulitis cultures). Some studies suggest that Gram-negative bacilli are the microorganisms most likely to cause bacteraemia or have a similar tendency to *S. aureus* to cause it [27, 28]. Other studies have also reported high rates of Gram-negative bacilli in blood cultures like the one by Gunderson et al. [19] (28%), which were even higher than that of *S. aureus*, as did Lee et al. [21] (24.2%). It is not surprising that this occurs for three reasons: as with *S. aureus,* it is also common to isolate these microorganisms from wounds and ulcers, particularly if they are of nosocomial origin [18] and if the patients are immunosuppressed [19, 21], have diabetes [20] or liver problems [21], as is the case in this study. In fact, challenging the traditional paradigm that does not routinely recommend Gram-negative coverage in cellulitis at least, in high-risk population [3, 19].

On the other hand, it should be noted that despite the increase in the presence of MRSA in skin and soft tissue infections worldwide [29, 30], only five cases have been isolated in this study, and only one have resulted in bacteremia.

In this regard, bacteremia not only indicates the presence of systemic infection but also the evolving dynamics of aetiological patterns, which may have implications for empirical treatment strategies, because nowadays, although there is no standardised treatment for cellulitis [31], there is a consensus on the use of antibiotics that cover *Staphylococcus aureus* and streptococci [9, 10, 18], dismissing Gram-negative bacilli [3, 21]. In any case, in this study, the most used antibiotics in cases of bacteremia are amoxicillin/clavulanic acid and piperacillin/tazobactam, antibiotics that cover the three most common pathogens in cellulitis bacteremia. The choice of these antibiotics is in line with the IDSA’s latest publication from 2014, in which it suggests that in this type of immunocompromised and/or seriously ill patient, broad-spectrum antibiotics such as piperacillin-tazobactam should be used [9].

Furthermore, the observed temporal variability, with a significant increase in incidence in recent years, especially in 2023, can be interpreted within a broader framework of post-pandemic epidemiological reconfiguration. A possible explanation is the so-called “immunity debt” generated during periods of reduced social interaction, which may have facilitated the resurgence and transmission of more virulent strains of *Streptococcus* species [32, 33]. This fact may suggest that epidemiological landscape of SSTIs is also evolving. Recent studies have suggested potential shifts in the incidence and microbiological profiles of invasive infections following the COVID-19 pandemic, yet long-term data spanning this transition are scarce [34, 35]. Additionally, delays in seeking medical care during the pandemic could have resulted in patients presenting with more advanced infections in the subsequent years, potentially increasing the likelihood of bloodstream involvement.

From a healthcare management perspective, bacteremia was associated with a significantly longer length of hospital stay and a higher frequency of antibiotic treatment modifications [6, 18]. First, treatment duration for bacteremia is longer than for uncomplicated cellulitis (14–21 days vs. 5–7 days), and second, the risk of bacteremia is associated with comorbid conditions that require closer monitoring and more complex therapeutic management [18]. If we add to these facts the possible presence of sepsis that affected 65.2% of our patients and which is associated to a poorer therapeutic response [36], the duration of treatment and hospital stay could be even longer.

In this study, mortality was primarily associated with older age, disease severity, and systemic involvement. It is known that patients older than 65 years with bacteremia are at a higher risk of mortality [24, 37], because of their more compromised immune systems. Furthermore, the increased risk of mortality when bacteremia is nosocomial or secondary is because these patients are usually hospitalized for another underlying condition, which often leads to immunosuppression or a weakened immune system. On the other hand, CREST classifications III and IV are the most severe and are most likely to lead to bacteremia so it is therefore logical that they result in higher mortality rates [2].

Finally, the mortality observed in the bacteremia group deserves particular attention. Although the difference did not reach statistical significance, the three-month all-cause mortality rate was notably higher among patients with bacteremia. This finding suggests that bacteremia in cellulitis may act as a surrogate marker of patient vulnerability [38], reflecting underlying frailty and comorbidity burden. Moreover, the persistence of mortality beyond hospital discharge highlights the importance of close clinical follow-up during the post-discharge period, which represents a particularly vulnerable phase for these patients, especially those who have suffered from bacteremia [39].

Overall, our findings have relevant clinical implications for the management of hospitalized patients with cellulitis. The identification of bacteremia as a marker of increased disease severity may support a more selective and risk-based diagnostic approach when interpreted alongside the overall clinical assessment rather than severity scores alone.

In addition, the observed association between bacteremia and poorer medium-term outcomes highlights the importance of careful post-discharge monitoring in this population. Future prospective studies are needed to validate clinical prediction models that integrate severity scores, comorbidities, and microbiological findings to better identify patients at risk of bloodstream infection and adverse outcomes. Furthermore, multicenter investigations would help clarify the evolving epidemiology of cellulitis-related bacteremia and optimize strategies for early diagnosis, antimicrobial stewardship, and long-term patient follow-up.

From a practical perspective, our findings suggest that blood cultures may be considered more selectively in patients with higher clinical severity, signs of systemic infection, or underlying immunosuppression. However, this approach should rely on comprehensive clinical assessment rather than severity scores in isolation.

### Strengths and limitations

This study has several limitations that should be acknowledged. First, its retrospective and single-center design may limit the generalizability of the findings to other healthcare settings or populations. Second, clinical documentation and follow-up data were obtained from electronic medical records, which may introduce information bias or incomplete data capture. Blood cultures were not systematically performed in all hospitalized patients with cellulitis, but rather at the discretion of the treating physician. Consequently, the study population likely represents a clinically selected subgroup with greater disease severity, which may limit the generalizability of the findings. One example of this is the length of stay, which is longer in patients with bacteremia, probably due to the other underlying conditions that most patients have. And finally, the prognostic factor model is based on all deaths among patients with bacteremia; it could not be established using only those patients who died from cellulitis-associated bacteremia, as there were too few of them (*n* = 13).

Despite these limitations, our study also has several important strengths. It represents one of the largest single-center cohorts evaluating bacteremia in hospitalized cellulitis patients with systematic microbiological characterization and long-term follow-up. The six-year study period allowed us to identify temporal trends and potential epidemiological shifts, particularly in the post-pandemic era. In addition, the integration of clinical severity scales, microbiological findings, treatment patterns, and medium-term outcomes provides a comprehensive overview of the clinical impact of bacteremia in cellulitis, offering valuable information for risk stratification and clinical decision-making in routine practice.

## Conclusion

Bacteremia in cellulitis is an infrequent but clinically relevant event associated with increased disease severity, longer hospitalization, and greater therapeutic complexity. Clinical severity assessment may contribute to identifying patients who could benefit from blood cultures and closer monitoring. However, because of the limited discriminatory performance observed, severity scores should be interpreted as adjunctive tools and not be used in isolation to guide clinical decision-making..

## Data Availability

No datasets were generated or analysed during the current study.
